# Comparison of femoral tunnel widening after anterior cruciate ligament reconstruction using cortical button fixation versus transfemoral cross-pin fixation: a systematic review and meta-analysis

**DOI:** 10.1186/s43019-020-0028-9

**Published:** 2020-01-29

**Authors:** Dae-Hee Lee, Dong-Wook Son, Yi-Rak Seo, In-Gyu Lee

**Affiliations:** 1 Department of Orthopaedic Surgery, Samsung Medical Center, Sungkyunkwan University School of Medicine, Seoul, Republic of Korea; 2 Department of Orthopaedic Surgery, Kangbuk Samsung Hospital, Sungkyunkwan University School of Medicine, 29 Saemunan-ro, Jongno-gu, Seoul, 03181 Republic of Korea

**Keywords:** Knee, Anterior cruciate ligament, Reconstruction, Femoral tunnel widening, Meta-analysis

## Abstract

**Background:**

The aim was to compare tunnel widening of autogenous hamstring anterior cruciate ligament reconstruction (ACLR) using cortical button versus cross-pin femoral fixation.

**Methods:**

The PubMed, Embase, and Cochrane Central Register of Controlled Trials databases were searched from inception to 11 April 2019. The study included all levels of evidence in studies that reported femoral tunnel widening and compared cortical button and cross-pin femoral fixation for ACLR.

**Results:**

Six studies were included, covering a total of 344 knees. Using transtibial techniques for ACLR, the mean absolute amount of femoral tunnel widening was significantly greater with cortical button fixation than with transfemoral cross-pin fixation (−0.30 mm; 95% confidence interval (CI) −0.56,−0.05 mm; *p*= 0.02). Using the transtibial technique, the mean relative percentage of femoral tunnel widening was significantly greater with cortical button fixation than with transfemoral cross pin fixation (−5.73%; 95% CI −10.32, −1.14% ; *p*= 0.01).

**Conclusion:**

The present meta-analysis revealed greater widening of the femoral tunnel when using cortical button fixation for hamstring ACLR via the transtibial technique than when using transfemoral cross-pin fixation.

## Introduction

Multiple options are available for femoral graft fixation in anterior cruciate ligament (ACL) reconstruction. Although several techniques are available, the best option for femoral fixation has not been established. The most common techniques for femoral fixation include cortical button fixation, transfemoral cross-pin fixation, and intra-osseous interference screws fixation.

Recent meta-analyses have concluded that cortical button femoral fixation for autogenous hamstring ACL reconstruction had no significant difference in terms of clinical outcomes and postoperative knee laxity compared with cross-pin femoral fixation [1, 2]. Although both cortical button and transfemoral cross-pin fixation for femoral graft have good clinical outcomes and postoperative knee laxity, a common complication after ACL reconstruction is bone tunnel widening. Bone tunnel widening is a frequent complication seen in ACL reconstructions using hamstrings; however, it remains unclear whether the enlargement of bone tunnels is correlated with poor clinical results [3–6]. The main impact of bone tunnel widening is on patients requiring revision surgery. A large bone tunnel may hinder the revision surgery and increase the need for bone grafts in a staged procedure [7].

The transfemoral cross-pin fixation system is positioned closer to the intra-articular opening of the tunnel than a cortical button system, which may decrease the mobility of the graft in the bone tunnel [3, 5]. Cortical button fixation allows for greater movement of the graft within the bone tunnel [6]. Therefore, the aim of this study was to determine whether the cortical button fixation involves more femoral widening caused by “the bungee and wind-shield wiper effects” than transfemoral cross-pin fixation. The present meta-analysis aimed to compare femoral tunnel widening in ACL reconstruction by cortical button fixation versus transfemoral cross pins. The hypothesis was that less widening of the femoral tunnel occurs when transfemoral cross pins are used compared with cortical button fixation.

## Materials and methods

### Literature search

This systematic review was conducted according to the Preferred reporting items for systematic reviews and meta-analyses (PRISMA) guidelines using a PRISMA checklist. Two reviewers independently conducted the search using the PubMed, Embase, and the Cochrane Central Register of Controlled Trials databases from inception to 11 April 2019. The electronic search citation algorithm used was as follows: (ACL OR anterior cruciate ligament reconstruction) AND (tunnel OR widening OR enlargement OR tunnel widening OR tunnel enlargement) AND (suspension OR suspensory OR EndoButton OR button OR TightRope OR Toggleloc OR transfemoral OR transcondylar OR Cross pin OR cross pins OR cross-pin OR Cross-pins or Rigidfix OR Transfix). Manual searches were also performed for articles potentially missed by the electronic search.

### Inclusion and exclusion criteria

The inclusion criteria for the analysis were that the study must have (1) included patients who underwent primary arthroscopic single-bundle ACL reconstruction, using soft-tissue grafts, with cortical button and cross-pin femoral fixation; (2) evaluated tunnel widening using validated imaging tools such as radiography, computed tomography (CT), and magnetic resonance imaging (MRI); and (3) had complete reporting of parameters including means, standard deviations, and sample numbers. The exclusion criteria were as follows: (1) studies of multiligament injuries or concomitant knee disorders that required surgery or revision surgery; (2) studies failing to clearly report the data that met our interest; and (3) biomechanical or animal studies. Disagreements on study selection were resolved by discussion and consensus between the 2 reviewers.

### Data extraction

The main focus of this study was the measurement of femoral tunnel widening in cortical button and transfemoral cross-pin fixation devices. Tunnel widening was measured by the change in femoral tunnel diameter compared to measurements by immediate postoperative imaging. The imaging tool was either CT or radiography. Tunnel widening was measured at various locations such as aperture, midportion, proximal portion, or the widest portion of the femoral tunnel. Therefore, we selected the most similar location of tunnel measurement to minimize heterogeneity around the midportion. The femoral tunnel width was measured at 1 cm from the aperture, 5 mm from the aperture, and at the midportion of the femoral tunnels at the joint. The measurement was performed perpendicular to the long axis of the tunnels. The tunnel widening was measured on the immediate postoperative radiographs and the last follow-up radiographs. If the immediate postoperative femoral tunnel diameter was not recorded, the drill reamer size was substituted for that data point. The absolute change in diameter of the femoral tunnel in millimeters and the relative change in percentage from the aperture to the midportion were measured.

### Assessment of methodological quality

The methodological quality of each study was assessed using the Newcastle–Ottawa Scale [8], as recommended by the Cochrane Non-Randomized Studies Methods Working Group. The Newcastle–Ottawa Scale’s star system awards stars depending on the level of bias. In the present study, the Newcastle–Ottawa Scale was adjusted to a scale that included only low, high, and unclear bias for our purposes. Each study was judged on three criteria: selection of the study groups, comparability of the groups, and ascertainment of either the exposure or outcome of interest for case–control or cohort studies, respectively. Any unresolved disagreements between reviewers were resolved by consensus or by consultation with a third investigator.

### Statistical analysis

The main outcomes of the meta-analysis were the mean differences in absolute and relative femoral tunnel widening according to fixation device in groups that underwent ACL reconstruction with transfemoral and extracortical fixation. Tunnel widening was compared with mean differences and 95% CIs. Heterogeneity was determined by estimating the proportion of between-study inconsistencies due to actual differences between studies, rather than differences due to random error or chance, using the I2 statistic with values of 25, 50, and 75% defined as low, moderate, and high, respectively. All statistical analyses were performed using RevMan version 5.3 and Stata/MP 13.0.

## Results

### Identification of studies

**Fig. 1:**
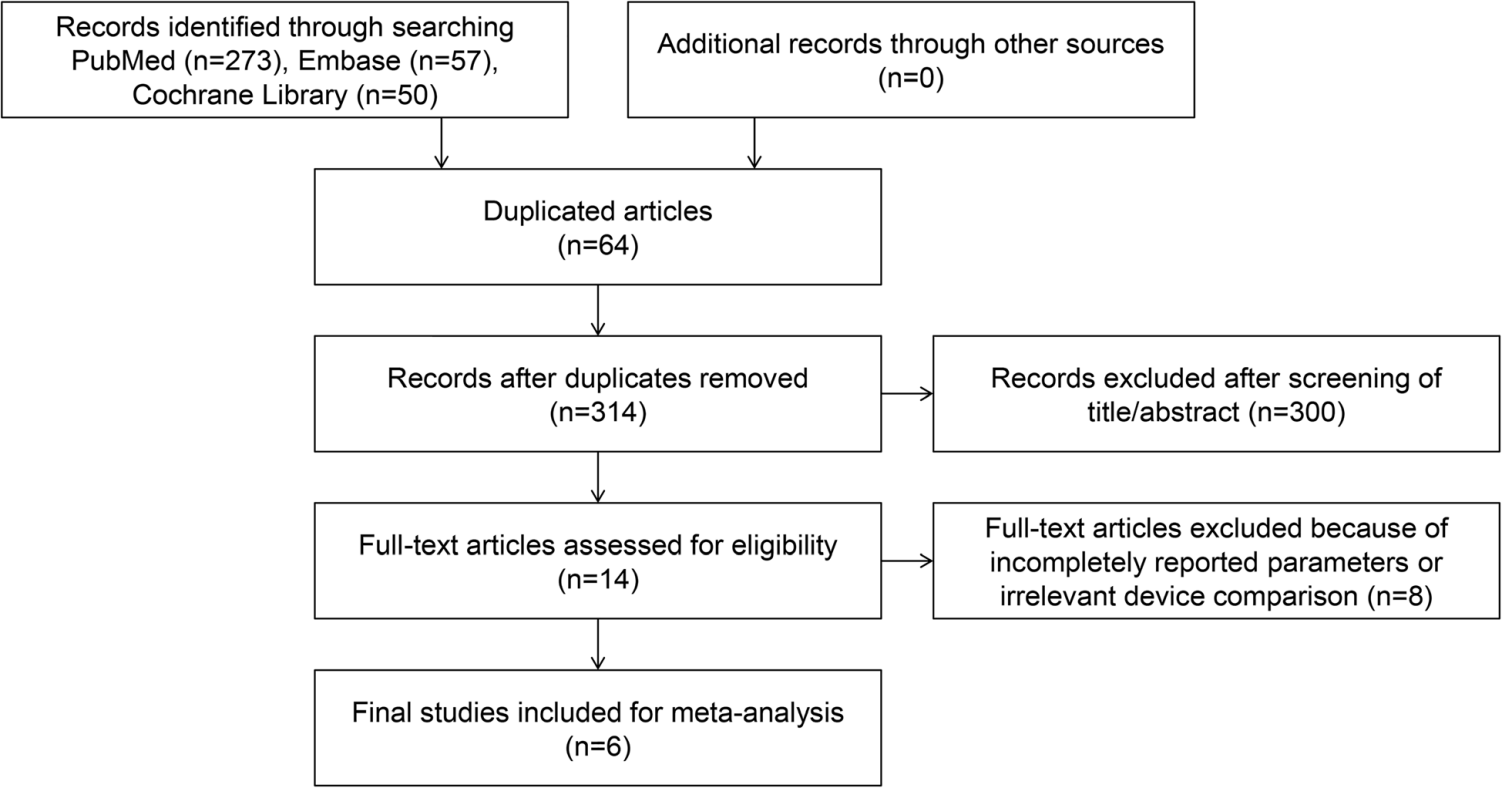
Preferred reporting items for systematic reviews and meta-analyses (PRISMA) flow diagram of the identification and selection of the studies included in this meta-analysis

After the search of PubMed, Embase, and the Cochrane Library, a total of 378 studies were included for screening. After removal of duplication, 314 articles remained. Through a review of titles and abstracts, the full text of 14 studies was retrieved for further screening. In total, six studies were included in this meta-analysis (Fig. 1). The six studies selected for inclusion covered 344 knees.

### Study characteristics and patient populations

The femoral fixation device, sample size, imaging tool, graft choice, and follow-up periods of the included studies are reported in Table 1. The six studies included 191 knees that underwent transfemoral fixation and 153 that underwent cortical suspensory fixation. Of the 191 knees undergoing transfemoral fixation, the anteromedial portal technique was used in procedures and the transtibial technique in 125; the technique was not mentioned for 17 procedures. Of the 153 knees that underwent extracortical suspensory fixation, the anteromedial portal technique was used in 23 procedures and the transtibial technique in 125; the technique was not mentioned for 5 procedures. Four studies retrospectively compared the absolute amount and the relative percentage of femoral tunnel widening, and two studies compared only the absolute amount of femoral tunnel widening.

**Table 1:** Characteristics of the studies included

Study	Year	Level of evidence	Technique	Imaging tool	Graft	Measurement level	Measurement method	Femur fixation device	Tibia fixation device	Sample size	Time of measurement (months)
Baumfeld et al. [9]	2008	IV	TT	x-ray	auto HT	1 cm from the aperture	Absolute change and relative percentage	Rigidfix	Intrafix	26	45.2
								EB	BIS + staple or screw and washer	20	41.8
Kong et al. [10]	2012	IV	TT	x-ray	auto HT	1 cm from the aperture	Absolute change	Rigid fix	Intrafix	56	57.4
								EB	BIS + metal screw and washer	35	55.5
Lopes et al. [11]	2017	III	AMP	CT	auto HT	5 mm from the aperture	Absolute change and relative percentage	Rigid fix	BIS	20	13.1
								EB	BIS	23	13.8
Saygi et al. [12]	2016	IV	TT	x-ray	auto HT	1 cm from the aperture	Absolute change and relative percentage	Cross-pin	BIS + staple	43	42.7
								Toggle Loc	BIS + staple	50	38.8
Srinivas et al. [13]	2016	II	NC	CT	auto HT	Mid-point	Absolute change	Cross-pin	Interference Screw or Suture Disc	17	12
								EB	Interference Screw or Suture Disc	5	12
Uzumcugil et al. [14]	2012	III	TT	x-ray	auto HT	1 cm from the aperture	Absolute change and relative percentage	Transfix	BIS + staple	29	29.4
								EB	BIS + staple	133	32.3

*AMP* Anteromedial portal, *BIS* Bioabsorbable interference screw, *EB* Endobutton, *HT* Hamstring tendon, *NC* Not commended, *TT* Transtibial

### Quality of the studies included

The studies included in this meta-analysis had a low risk of selection bias. One study did not compare demographic data between the groups undergoing cortical button and transfemoral cross-pin fixation, and none assessed possible confounding factors. Follow-up duration was defined as the time interval between surgery and the measurement of tunnel widening on CT or radiography. If postoperative femoral tunnel widening on CT or radiography was measured within 1 year after surgery, the studies was considered at high risk of bias. The risk of bias in the six studies included is summarized in Table 2.

**Table 2:** Risk of bias summary: review of authors’ judgments about the risk of bias in each study included

References	Representativeness of the cases	Selection of control	Ascertainment of exposure	Interest outcome not present at start cohorts of study	Comparability of cohorts	Control for any additional factors	Assessment of outcome	Enough follow up	Adequacy of follow up
Baumfeld et al. [9]	–	–	–	–	+	+	+	+	–
Kong et al. [10]	–	–	–	–	+	+	+	+	–
Lopes et al. [11]	–	–	–	–	–	+	+	+	–
Saygi et al. [12]	–	–	–	–	+	+	+	+	–
Srinivas et al. [13]	–	–	–	–	+	+	+	–	–
Uzumcugil et al. [14]	–	–	–	–	+	+	+	+	–

+ low risk of bias, − high risk of bias

### Absolute amount of femoral tunnel widening

**Fig. 2:**
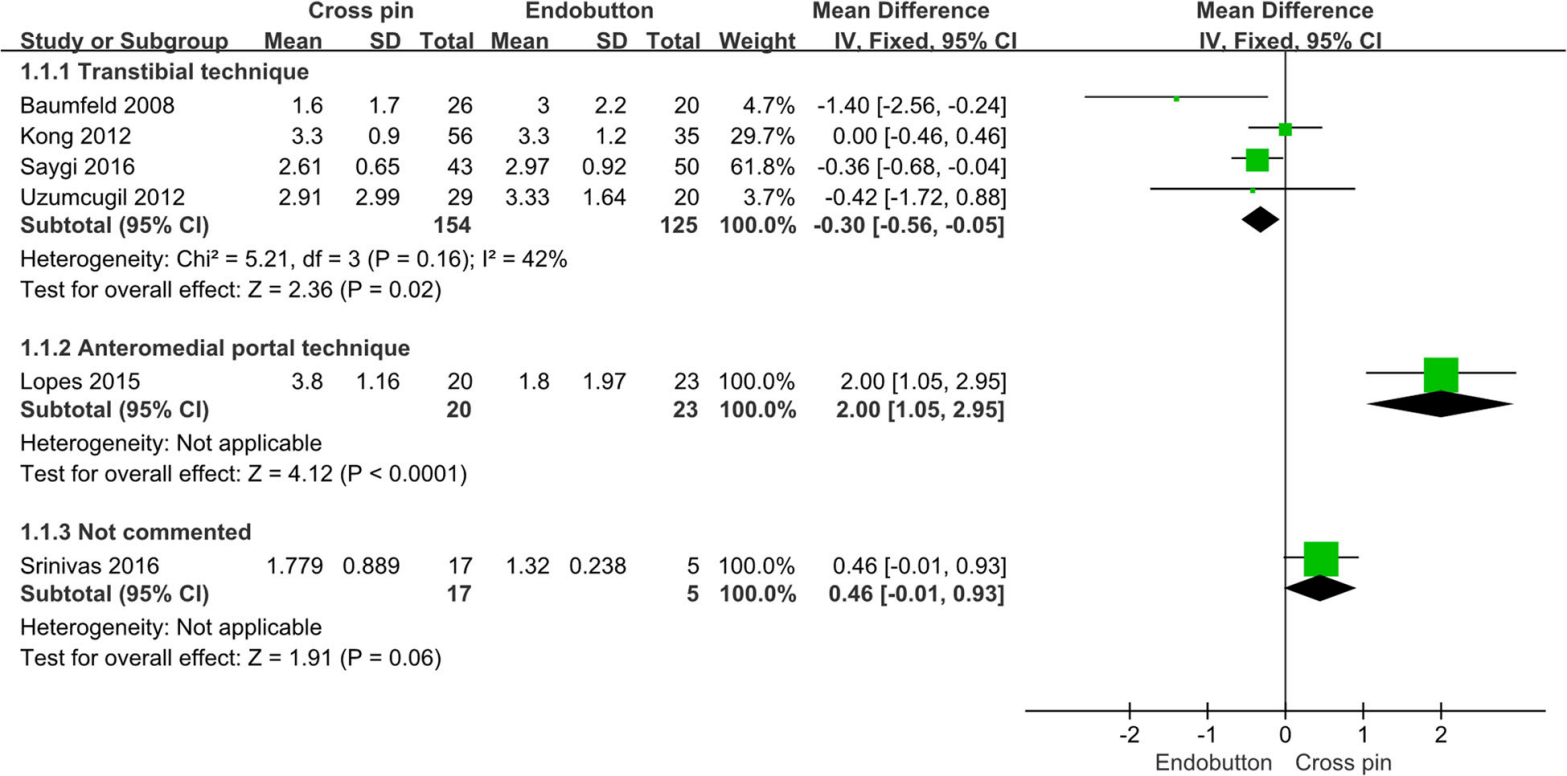
Absolute amount of femoral tunnel widening in cortical button versus cross-pin fixation according to different surgical techniques

All six studies reported absolute change in tunnel diameter and included 191 and 153 knees that underwent ACL reconstructions by cortical button and transfemoral cross-pin technique, respectively. Using transtibial techniques for ACL reconstruction, the mean absolute amount of femoral tunnel widening was found to be significantly greater for cortical button fixation than transfemoral cross-pin fixation (−0.30 mm; 95% CI − 0.56, − 0.05 mm; *p* = 0.02, Fig. 2). One study using an anteromedial portal technique for ACL reconstruction showed that femoral tunnel widening was 2.0 mm grater in transfemoral cross-pin fixation. The overall mean absolute amount of femoral tunnel widening was not provided because of potential selection bias. There was only one study using the anteromedial portal technique and the report of one study did not comment on the femoral tunnel drilling technique.

### Relative percentage of femoral tunnel widening

**Fig. 3:**
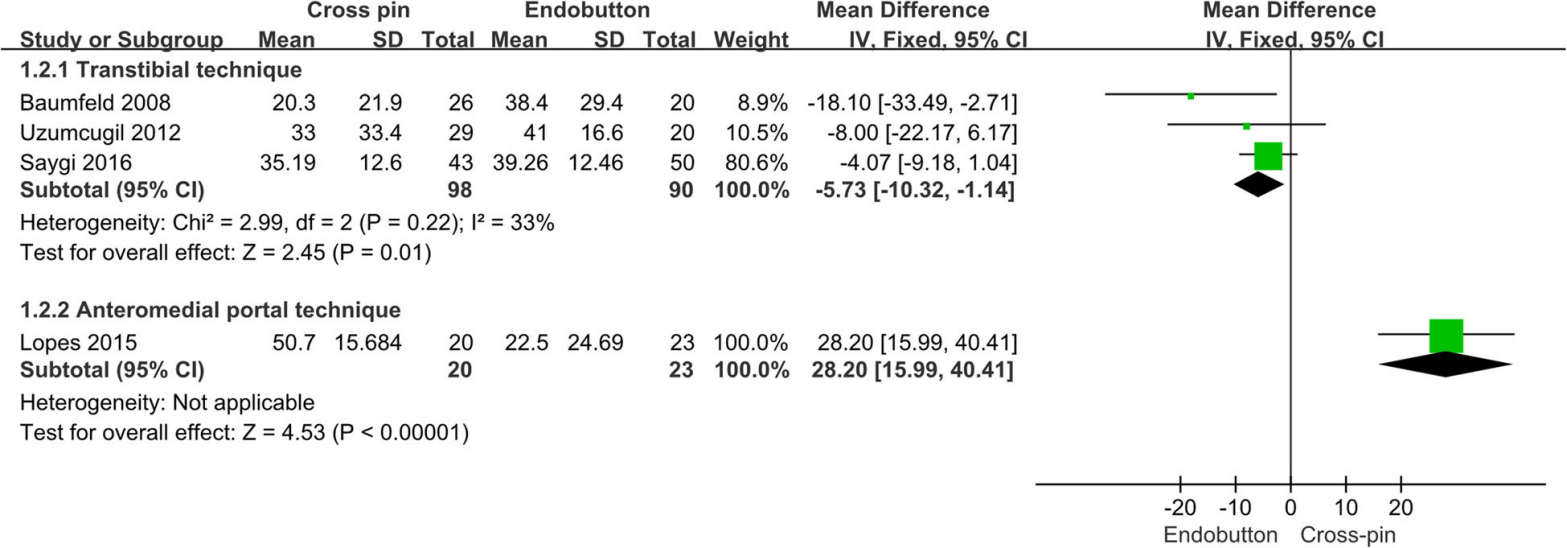
Relative percentages of femoral tunnel widening in cortical button versus transfemoral cross-pin fixation according to different surgical techniques

Of the six studies, four compared relative percentage of femoral tunnel widening between cortical button fixation and transfemoral cross-pin fixation. In total, 118 patients underwent transfemoral cross-pin fixation and 113 underwent cortical button fixation. Using the transtibial technique, the mean relative percentage of femoral tunnel widening was significantly greater for cortical button fixation than for transfemoral cross pin fixation (−5.73%; 95% CI −10.32,−1.14%; *p* = 0.01, Fig. 3). One study using an anteromedial portal technique for ACL reconstruction did not show a significantly different mean relative percentage of femoral tunnel widening between the two groups.

### Absolute amount of tibial tunnel widening

**Fig. 4:**
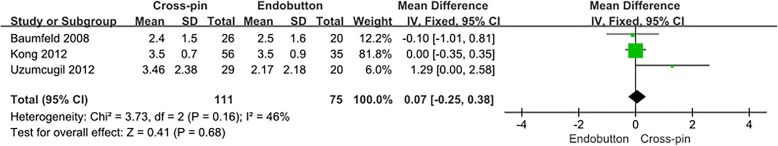
Absolute amount of tibial tunnel widening in cortical button versus transfemoral cross-pin fixation

Of the six studies, three compared the absolute amount of tibial tunnel widening between cortical button fixation and transfemoral cross-pin fixation. In total, 111 patients underwent transfemoral cross-pin fixation and 75 underwent cortical button fixation. There was no significant difference in mean absolute change between transfemoral cross-pin and cortical button fixation (0.07 mm; 95% CI − 0.25, 0.38mm; *p* = 0.68, Fig. 4).

## Discussion

On the basis of our definition of tunnel widening, the most important finding in the present study was that femoral tunnel widening in cortical button fixation was greater than transfemoral cross-pin fixation, when the transtibial technique was used. Therefore, cortical button fixation is associated with slightly more femoral widening caused by “the bungee and wind-shield wiper effects” than transfemoral cross-pin fixation using transtibial technique. However, there was a small difference in femoral tunnel widening in terms of the absolute amount of change, and one study using the anteromedial portal technique showed significant widening in transfemoral cross-pin fixation. Therefore, further studies are needed to provide enough clinical evidence for the femoral tunnel widening in ACLR.

Tunnel widening after ACL reconstruction is caused by various mechanical and biological factors. The difference in the graft fixation device is one such factor. Some authors report that cortical button fixation increases the movement of the graft inside the bone tunnel and at the intra-articular aperture, which are known as the bungee effect and the wind-shield wiper effect, respectively [15]. If the distance of the cortical fixation device from the joint space is decreased, the shorter tunnels could reduce these effects [2]. Another possible factor is tunnel placement. If the graft-bending angle of ACL reconstruction is more acute at the aperture of the tunnel, this could induce higher stress on the graft-bone interface. Biological factors in tunnel widening include bioabsorbable material for graft fixation and extravasations of synovial fluid. The decomposition of bioabsorbable screws could promote tunnel widening because of a chemical osmotic effect inside the bone tunnel [16, 17].

Reports from several studies suggest that femoral tunnel widening may be significantly lower when fixation takes place closer to the joint. If fixation is far from the joint, the bungee and wind-shield wiper effects could be magnified [2]. Fauno and Kaalund [6] measured tunnel diameters using radiographs at points 10 mm away from the aperture and found that both femoral and tibial tunnel widening occurred significantly more frequently in the cortical button group than in the transfemoral cross-pin group. Sabat et al. [18] measured tunnel enlargement using CT at the aperture, midway, and suspension points. They reported that tunnel widening was significantly less in the transfix group than in the EndoButton group. Saygi et al. [12] investigated whether the tight-fit technique decreases tunnel widening and improves clinical outcomes. Their study included a total of 93 patients who underwent hamstring tendon ACL reconstruction using either cortical-cancellous suspension (Cross pin, Biomet, USA) or cortical suspension (ToggleLoc, Biomet, USA) for femoral fixation with 7 mm and 8 mm tunnel diameter. They reported that button fixation was associated with greater tunnel widening, as shown previously in the literature. However, they concluded that undersize-drilled cases of button fixation were associated with the least femoral tunnel widening and also the best clinical scores among all cases included in the study. They insisted that this was caused by tight fixation of the hamstring tendon graft, which decreased the graft–tunnel motion. Baumfeld et al. [9] compared femoral and tibial tunnel widening and functional outcomes between double cross-pin fixation and suspensory fixation following hamstring autograft ACL reconstruction with a minimum of 2 years of follow up. One group used suspensory femoral fixation (EndoButton) and routinely used the Intrafix device for tibial fixation; the other used femoral cross-pin fixation (Rigidfix) and a bioabsorbable interference screw with some form of back-up fixation. They reported that there was significantly more femoral tunnel widening associated with the use of the EndoButton suspensory fixation system compared to the use of double cross-pins fixation within the tunnel.

Other studies reported that femoral tunnel widening was not significantly different even though fixation takes place closer to the joint. Kuskucu [19] compared the results of two femoral fixation methods (cross-pin and EndoButton™ CL) with respect to tunnel enlargement. They measured the diameters of both femoral and tibial tunnels at three different points from the aperture with 5-mm intervals on standard anteroposterior and lateral radiographs after 12 months. They reported that a significant difference in tunnel widening was detected between groups only on the tibial side; however, they only compared relative percentage of tunnel widening between groups. Kong et al. [10] retrospectively compared the clinical and radiological results of EndoButton femoral fixation for reconstruction with those of cross-pin fixation. One hundred twenty-six autogenous hamstring ACL reconstructions were performed using either cross pins or EndoButton CL for femoral fixation with an 8-mm diameter tunnel width. They reported that there were no significant differences in the amount of femoral and tibial tunnel widening between the groups. They concluded that both cross-pin and EndoButton CL are useful materials for femoral tunnel fixation in hamstring ACL reconstruction surgery. Uzumcugil et al. [14] evaluated the effect of the AperFix device (Cayenne Medical, Inc., Scottsdale, Arizona), composed of polyetheretherketone (PEEK) polymer, on tunnel widening after hamstring anterior cruciate ligament reconstruction as compared with two other fixation devices: TransFix (Arthrex, Inc., Naples, FL, USA) and EndoButton (Smith & Nephew Endoscopy, Mansfield, MA, USA). They reported that tunnel widening between groups was not significantly different in terms of coronal and sagittal femoral tunnel diameters, and tibial tunnel diameter increase in the sagittal plane in the EndoButton group was significantly smaller than that in the TransFix and AperFix groups. They suggested that tunnel enlargement after ACL reconstruction is influenced by the type of graft fixation only on the tibial side irrespective of clinical outcome. Lopes et al. [11] showed greater enlargement of the femoral bone tunnel when a bioabsorbable trans-tunnel pin system was used with the medial portal technique when compared to extracortical fixation. They suggested that the absorbable material that composes the RigidFix and the soft tissue graft used may contribute to the development of femoral tunnel enlargement.

The present study has several limitations. First, the imaging tool used to measure the diameter of the tunnel was different depending on the study. Plain radiography or CT were used for measurement in all studies; however, conventional radiography showed good correlations with CT and MRI imaging. Second, the location of measurement of tunnel widening differed in each study included. Femoral tunnel widening was measured at various locations such as the aperture, mid-portion, and suspension point. Therefore, we selected the most similar location of tunnel measurement to minimize heterogeneity. Third, the anteromedial technique of femoral drilling was used only in one study. Therefore, we could not perform meta-analysis to compare the cortical button and transfemoral cross-pin fixation using the anteromedial technique. Fourth, the morphology of femoral tunnel widening was not described in the studies included. The morphologic difference in tunnel widening at the aperture and proximal end could be attributed to the windshield-wiper effect.

## Conclusions

The present meta-analysis revealed greater widening of the femoral tunnel when using cortical button fixation with the transtibial technique than when using transfemoral cross-pin fixation. Cortical button fixation is associated with slightly more femoral widening caused by the bungee and wind-shield wiper effects than transfemoral cross-pin fixation using the transtibial technique.

## Data Availability

Not applicable.

## Abbreviations

ACL: Anterior cruciate ligament
ACLR: Anterior cruciate ligament reconstruction
CT: Computed tomography
MRI: Magnetic resonance imaging
PRISM: Preferred reporting items for systematic reviews and meta-analyses
